# Supplementary material for: Are carbon emissions trading and green financial instruments synergistic? -Comprehensive quantitative research based on content analysis
Source: PLoS One. 2024 Mar 7;19(3):e0298601. doi: 10.1371/journal.pone.0298601 (PMC10919601; doi:10.1371/journal.pone.0298601)
Supplement: S1 Data — (ZIP) [file pone.0298601.s001.zip › Data/地级市Z_F工作报告.zip!/╜¡╦╒/╬▐╬2╩╨/└┤╫╘╣1⁄2╓┌║┼í╛┬φ┐╦╩2╛▌═°í┐_2015.docx]

各位代表：

　　现在，我代表无锡市人民政府向大会报告工作，请予审议，并请市政协委员和其他列席人员提出意见。

　　2014年工作回顾

　　2014年是全面深化改革的开局之年。过去的一年，面对严峻复杂的宏观形势和艰巨繁重的改革发展稳定任务，在中共无锡市委的正确领导下，全市上下认真学习贯彻党的十八大、十八届三中、四中全会和习近平总书记系列重要讲话精神，扎实开展党的群众路线教育实践活动，坚持稳中求进、深化改革创新，统筹做好稳增长、促改革、调结构、重生态、惠民生、防风险各项工作，经济社会保持平稳健康发展。预计全市地区生产总值同比增长8.2%，一般公共预算收入增长8.0%，城乡居民人均可支配收入分别增长9%和10%，城镇登记失业率1.91%，居民消费价格指数102.2，基本完成市十五届人大三次会议确定的目标任务。

　　（一）全面深化改革成效显著

　　坚持以全面深化改革统领全局，围绕中央和省、市委关于改革工作的战略部署，下好改革“先手棋”，抓好具体改革举措落实，重点领域和关键环节改革取得实质性进展。

　　以简政放权为突破口，着力推进行政审批制度改革，政府职能转变力度进一步加大。承接落实国家和省下放取消的行政审批事项，取消和下放市级行政审批事项214项，完成市级非行政许可审批事项的清理工作，建立并向社会公布市级政府权力清单。在全省率先启动实施注册资本登记制度改革，新登记内资企业数和注册资本同比增长30.6%和121.9%。加快政务服务管理办公室及政务服务中心、公共资源交易中心、政府公共服务热线受理中心“一办三中心”体系建设。推进行政审批服务监管流程再造，减少审批环节606个，实际办结时限提速51%以上。

　　以激发市场主体活力为导向，着力推进经济体制改革，资源配置方式进一步优化。深化民营经济改革，放宽民间资本市场准入，积极引进民间资本参与基础类、公益类项目建设。稳步推进国资国企改革，积极发展混合所有制经济，17个国有企业项目吸收非国有资本参股投资12.3亿元。加快推进财税体制改革，建立全口径预算管理体系，扎实做好“营改增”试点扩围工作。深化农村产权制度改革和集体资产股份制改革，全市84个村开展土地承包经营权确权登记颁证试点，30个村完成集体资产股份合作改革。稳步推进水、天然气等资源环境价格改革。创新科技计划项目管理机制，完善科技金融体系，科技型中小企业贷款风险补偿资金池累计放款28.3亿元。加快发展地方金融机构，无锡农商行江阴、宜兴支行开业，互联网金融“开鑫贷”公司落户无锡，国联人寿、三房巷集团财务公司、全国首家物联网产品电子交易中心、省内首家市级金融资产交易中心获批筹建，海峡两岸金融与科技合作试验区获省政府批准。引进外资商业保理公司2家、融资租赁公司11家，东亚银行无锡分行开业。

　　以增创开放型经济新优势为目标，着力推进对外开放体制机制创新，以开放促改革、增活力，城市国际化水平进一步提高。全面对接上海自贸区，设立驻自贸区代表处，复制推广33项改革事项，推行口岸无障碍通关，启用世贸通中小微外贸企业综合服务平台，探索试行多项外汇管理便利化措施。无锡航空口岸扩大对外国籍飞机开放，开通至首尔国际航线，韩亚航空进驻无锡，国际快件中心正式投用，苏南硕放国际机场旅客吞吐量418万人次、货邮吞吐量9.6万吨。加强对外交流合作，新增国际友城2个，荣获“国际友好城市交流合作奖”。

　　以增加公共服务供给为重点，着力推进社会领域改革，民生保障机制进一步完善。深化文化体制改革，创新文化产品供给服务机制。全面实施县级公立医院综合改革，完善分级诊疗体系。优化养老服务业发展政策环境，开展全国养老服务业综合改革试点。顺利实施单独两孩政策。优化调整户口准入政策，在全国率先实现大市范围内户籍准入登记、迁移规定一体化。

　　（二）经济运行质量稳步提升

　　面对经济下行压力，认真贯彻落实国家、省一系列稳增长政策，结合实际出台相关配套措施，主攻薄弱环节和重点领域，强化资源保障和协调服务，有效推动经济稳定增长。完成固定资产投资增长16%；社会消费品零售总额增长11.5%；进出口、出口总额分别增长5.4%、7.5%。发挥企业稳增长主力军作用，着力稳定工业运行，规模以上工业增加值增长4.9%，预计规模以上工业企业利税和利润增速分别提高2.4和6.5个百分点。继续推动百企做强，15家企业入围2014中国企业五百强，连续八年居全省首位。积极优化金融服务，扩大有效信贷规模，实现社会融资规模1410亿元，其中新增人民币贷款561.5亿元；新增境内外上市企业4家、新三板挂牌企业37家，新增区域性股权交易中心挂牌企业21家、各类私募股权基金12家。高度重视防范和化解金融风险，建立中小企业转贷应急资金平台，全市累计为2006家企业提供转贷应急资金377.5亿元，钢贸行业债务风险基本化解，银行业金融机构不良贷款余额和不良率同比实现“双下降”。

　　在稳增长基础上，加大结构调整力度，促进转型升级向纵深推进。鼓励战略性新兴产业发展，着力提升规模层次，预计新兴产业产值增长15%，高新技术产业产值占规模以上工业总产值比重提高0.5个百分点。国家传感网创新示范区建设成效显著，物联网及相关产业业务收入增长40%以上，一批物联网应用示范项目获全国推广，无锡连续三年获中国智慧城市发展评估第一名，入选国家级“两化”深度融合试验城市。推动文化创意、旅游休闲、会展经济、电子商务、健康养老等现代服务业加快发展，成为国家信息惠民试点城市和电子商务示范城市，苏南快递产业园获批全国快递产业集聚发展试验园区，新增国家4A级景区5家，无锡城市游客满意度居全国第一，预计服务业增加值占地区生产总值比重达47%，同比提高1个百分点。完善现代农业产业体系，新增高标准农田2万亩、高效设施农业（渔业）3万亩，农业园区化比重达40%以上，率先实现农业现代化建设取得新进展。

　　实施创新驱动战略，加快推动科技创新创业，不断提升科技和人才竞争力。推进“人才强企”工程，累计引进9名诺贝尔奖得主、11名外籍院士与民营企业共建研究院，成立“欧美同学会”留学报国无锡基地。预计全社会研发投入占地区生产总值比重达2.75%；万人有效发明专利拥有量超过18.3件，同比增长23 %，国家专利奖获奖数列全国地级市首位。兴澄特钢获得“全国质量奖”，红豆集团获评“全国质量标杆企业”。无锡高新区（含宜兴环科园）、江阴高新区入列苏南国家自主创新示范区。无锡获评福布斯“中国大陆最佳商业城市”第四名。

　　（三）重大项目建设扎实推进

　　开展“项目建设深化年”活动，持续实施项目攻坚，投资和重大项目建设实现新突破。围绕全年投资计划，重点实施180个事关后劲培育、民生改善和城市发展的重大项目，当年完成投资超1000亿元。充分调动企业投资积极性，全市民间投资占固定资产投资比重达63.1%；完成工业投资1746亿元，增长12.5%，实施“千企技改”项目1593个。强化与央企、沪企等战略合作，加快签约项目的跟踪推进，努力化解土地、资金等要素制约，重大项目建设成效进一步显现。中芯国际集成芯片、华泰汽车自动变速器等一批超50亿元的项目开工建设；海力士五期、万达文旅城、世茂旅游等一批超百亿元的重特大项目进展较好；高德印刷电路板、隆基硅材料超薄单晶硅、红牛饮料二期等一批重大工业项目竣工投产。城市基础设施项目顺利实施，地铁1、2号线开通试运营，苏南硕放国际机场二期航站楼竣工试运行，北中路互通、广南路、环山西路、江阴大道、海港大道等一批道路建成通车，锡澄运河江阴段航道工程基本完成。积极推进“一城一岛一带”建设，海岸城、万象城建成开业，巡塘古镇、灵山小镇一期、古运河绿地景观和亮化工程基本完工，重点区域功能配套不断完善。着力转变利用外资方式，提高利用外资质量，突出先进制造业和现代服务业重点，共引进3000万美元以上重大外资项目64个，英飞凌半导体、博世汽车动力总成等一批投资超亿美元重大产业项目成功签约，预计完成到位注册外资31亿美元，其中制造业占比达55%以上。

　　（四）城乡生态环境持续改善

　　深入实施“两型社会”综合配套改革试点，全面推进污染治理和环境整治，着力构建生态文明体系，城市宜居品质不断提升。开展新一轮太湖治理，扎实推进控源截污、生态清淤、蓝藻打捞、调水引流等工作，太湖无锡水域水质稳定好转，连续七年实现安全度夏。深入实施“蓝天工程”，突出抓好燃煤锅炉整治和工业废气、工地扬尘、机动车尾气、秸秆焚烧等污染防治，大力实施热网整合工程，PM2.5年均浓度下降幅度超额完成国家考核任务。启动锡钢地块土壤修复工程。严厉打击环境违法行为，查处环境违法案件878件，否决、劝退不符合环保要求项目79个。全面完成淘汰落后产能和化解过剩产能年度任务，加大节能减排力度，预计万元地区生产总值能耗下降4%以上，化学需氧量、二氧化硫、氨氮、氮氧化物排放削减量均超额完成省定目标。制定实施主体功能区计划，划定生态红线保护区域，推进土地节约集约利用，建立盘活存量和使用增量挂钩制度，预计单位建设用地产出继续保持全省第一。健全生态文明制度，建立生态补偿机制，继续实施排污权有偿使用和交易制度、推行环境污染责任保险，设立全国首个地级市环保公益发展基金。

　　制定实施城市现代化和城乡发展一体化规划，积极稳妥推进新型城镇化。推动城市重点功能板块建设，促进特色发展与产城融合。加快中心城区更新，增加旧住宅区改造实施计划，整治改造城中村、旧住宅区、危旧住房分别达16.6万、205万和10.3万平方米。强化环境综合整治，市区拆除违法建筑26.9万平方米，完成17条主要道路包装出新和56个背街小巷综合治理，推进147个生活垃圾分类收集、分类处置试点。加大植树造林力度，完成造林绿化2万亩，林木覆盖率预计达26.5%。新增8个城乡发展一体化先导示范镇，宜兴白塔村、惠山阳山村、滨湖古竹社区被评为江苏最具魅力休闲乡村。江阴新桥镇新型社区建设获国家人居环境范例奖，宜兴周铁镇成为中国历史文化名镇，荡口古镇获中国最美村镇人文环境奖。无锡获评全国首批创建生态文明典范城市、中国宜居城市。

　　（五）为民办实事项目圆满完成

　　把保障和改善民生放在优先位置，加大财政投入，精心组织实施，12件79项为民办实事项目全面完成。鼓励支持就业创业，全市新增城镇就业14.9万人，其中新增大学生就业4.1万人，扶持自主创业1.2万人。扎实推进社会保障扩面提标和新老制度衔接，城乡基本养老、医疗、失业保险覆盖率均超过98%，市区居民基础养老金、企业退休人员养老金、低保家庭中的重度残疾人救助标准分别提高到每月340元、2230元和792元，市区城乡低保标准提高到每人每月660元，全市最低工资标准提高到每月1630元。在全省率先实施城乡居民大病保险制度，4.5万人次获大病补偿9100万元，职工、居民医保政策范围内住院费用报销比例稳定在80%和70%以上。拓宽“慈福”保险领域，在市区和江阴实施户籍居民住房财产保险制度，安康关爱保险受益老年人48.38万人。支持社会化养老机构建设，新增机构养老床位3009张，居家养老服务站基本实现城市社区全覆盖、农村社区覆盖率达90%。改善低收入居民居住条件，全市新开工、筹集各类保障性住房5186套，发放廉租房租赁补贴2397户。加强常规公交与轨道交通有效衔接，市区新辟公交线路12条、优化调整61条。完善城市生活配套设施，改造老旧自来水管网64.7千米，新增天然气用户8.3万户，改造老新村天然气7900户，一流配电网建成区面积836平方千米，新增免费无线接入点2.7万个。新建、改造农贸市场20家，培育建成放心粮油单位85家，214家平价商店累计惠民1.7亿元。

　　（六）各项社会事业全面发展

　　繁荣发展社会事业。加快教育现代化建设，完善终身教育体系，全市教育现代化水平位列全省第二。深入推进义务教育均衡发展，全面提升初中教育质量水平，90%以上义务教育学校达到省定现代化办学标准。新建和改扩建幼儿园36所、社会化早教机构23所，建成15个市学前教育现代化镇（街道）。深化职业教育综合改革，组织实施省现代职教体系建设试点项目45个。深化与江南大学共建合作，支持民办院校创新发展。加快基层医疗卫生服务机构提档升级，新建、改建社区卫生服务中心16家，全面完成社区卫生服务站标准化建设。推进家庭医生制度，6类重点人群健康管理服务实现全覆盖。积极创建第二批国家公共文化服务体系示范区，顺利通过国家中期督查，公共文化设施免费开放范围不断扩大，“书香无锡”全民阅读活动全面开展。大运河无锡段参与申遗成功，惠山祠堂群列入省2016年推荐申遗项目，“宜兴紫砂陶制作技艺”申报世界非遗正式启动，“泰伯庙会”“均陶制作技艺”被评为第四批国家级非遗代表性项目，紫砂文化海峡两岸交流基地创建成功。大力弘扬社会主义核心价值观，全国文明城市创建通过省级测评。成功举办国际马拉松赛、世界斯诺克无锡精英赛、环太湖国际公路自行车赛等赛事，圆满完成第18届省运会参赛任务。建成西蠡湖慢游系统全民健身带，“10分钟体育健身圈” 基本建成。江南大学无锡医学院及其附属医院、市五院、市少年宫、太湖新城国际教育园等一批社会事业重大项目有序推进。

　　加强和创新基层社会治理。开展社区“减负增效”，全面清理面向社区的创建评比、机构挂牌、台账目录，理清村（居）民委员会依法履职事项和协助政府工作事项两份清单，全市65个镇（街道）试点“政社互动”。改革社会组织登记制度，全市社会组织达11576个。继续开展公益创投等活动，推动社会组织参与社会公益服务和社区治理。扎实推进立体化社会治安防控体系建设，严密防范打击暴恐犯罪，圆满完成青奥安保各项任务，社会治安保持稳定。加强食品药品安全监管，食品、药品安全检测合格率分别达97.4%和98.6%。完善应急体系网络，加强应急能力建设，及时有效处置各类突发事件。严格落实安全生产“党政同责、一岗双责”，集中开展安全生产检查整改专项行动，全年未发生重特大安全生产事故。创新信访工作体制机制，深入排查矛盾纠纷，有效化解一批信访积案。国防后备力量建设和军民融合式发展走在全省前列。史志、档案、侨务、台港澳、外事、民族宗教、人武、人防、气象、防汛防台、防震减灾等工作取得新成绩，妇女、儿童、红十字、老龄、残疾人等事业有了新发展。

　　（七）政府自身建设得到加强

　　围绕建设人民满意政府，强化宗旨意识，转变工作作风，服务发展能力进一步提升。扎实开展党的群众路线教育实践活动，坚决落实中央八项规定和省、市委十项规定，深入推进“作风建设深化年”活动，集中整改“四风”突出问题，政风建设取得积极成效，以市政府名义召开的会议和下发的文件分别减少26%和18%。健全政府工作规则，规范政府性投资项目决策程序，重大事项按规定程序集体讨论、民主决策。坚持依法行政，提交地方性法规议案5件，出台市政府规章6件、规范性文件22件。认真贯彻市人民代表大会及其常委会的各项决议，定期向市人大报告工作、向市政协通报情况，扎实做好代表建议和委员提案办理工作。做好“十二五”规划中期评估，圆满完成第三次全国经济普查。加强政务公开，政府预决算及“三公”经费决算向社会公开，无锡政府门户网站连续四年蝉联全国地市级第一，“无锡发布”被评为十佳城市政务新媒体。强化政府性债务管理，建立偿债准备金制度，政府性债务压规模、降成本、控风险取得明显成效。加大反腐倡廉力度，严肃查处一批违法违纪案件。

　　各位代表，这些成绩来之不易，凝聚着全市人民的智慧和汗水。在此，我代表无锡市人民政府，向在各个领域和不同岗位上辛勤劳动、无私奉献的全体市民，向辛勤工作在村组、社区、企业等基层组织和单位的广大干部员工，向给予政府工作积极支持的人大代表和政协委员，向离退休老同志，向各民主党派、工商联、无党派知识分子联谊会、各人民团体和各界人士，向中央及省在锡单位，向驻锡人民解放军、武警官兵和公安干警，向所有参与、支持和关心无锡建设发展的海内外朋友，表示崇高的敬意和衷心的感谢！

　　各位代表，我们清醒地看到，我市经济社会发展中还存在一些问题和矛盾，政府工作也存在一些不足。全面深化改革成效有待进一步释放，经济社会发展活力尚未充分激发；部分行业和企业运行困难，少数经济指标未能完成预期目标；产业结构仍然处于产业链条的中低端，新兴产业对经济增长的支撑不够有力，经济提质增效的基础不够牢固；政府性债务、房地产、金融等领域仍然存在风险隐患；大气、水污染和垃圾处理等环境治理任务依然艰巨；有的政府部门和工作人员服务意识和办事效率不高，反腐倡廉力度需要进一步加大。我们将对此高度重视，在今后工作中认真加以改进。

　　2015年工作任务

　　今年是全面深化改革的关键之年，是全面推进依法治国的开局之年，也是全面完成“十二五”规划的收官之年。做好今年政府工作的总体要求是：深入贯彻党的十八大和十八届三中、四中全会精神，认真落实习近平总书记对江苏工作的最新要求，按照省委、省政府和市委的决策部署，坚持稳中求进工作总基调，主动适应经济发展新常态，深入开展项目建设、改革创新、作风建设“三个突破年”活动，把“迈上新台阶、建设新无锡”鲜明地写在无锡发展的旗帜上，把转方式调结构和生态文明建设放在更加重要位置，把新型城镇化和民生保障的事情办好办实，把全面深化改革和全面推进法治建设贯穿于政府工作全过程，切实加强服务型政府和廉洁政府建设，奋力开创“四个无锡”和率先基本实现现代化建设新局面。

　　今年经济社会发展主要预期目标是：地区生产总值增长8%左右；一般公共预算收入增长8%左右；城镇登记失业率控制在3.5%以内；居民消费价格指数控制在省定范围内；万元地区生产总值能耗和主要污染物排放削减量完成省下达指标。上述主要预期目标，既考虑到新常态下保持一定的增长速度，又为深化改革、调整结构留出了空间。

　　做好今年工作，必须充分认识、主动适应经济发展新常态。当前，无锡和全国一样处于“三期叠加”阶段，正在经历消费、投资、出口三大需求的深刻变化，承受资源环境脆弱、经济风险积累的压力，面临生产组织方式、经济发展动力的转换，经济发展已经从高速增长转向中高速增长。应当清醒认识到，新常态下保持经济中高速增长不是自然而然可以实现的，必须作出不懈努力，稳增长仍然是今年经济工作的重要任务。对于无锡来说，遇到的困难、风险和挑战要更大一些、更早一些，保持经济稳定增长需要加倍努力、付出更多。我们对当前经济运行和改革发展中的困难挑战不能无所作为，对宏观环境变化带来的工作压力不能有畏难情绪，必须保持昂扬向上、勇于拼搏的精神状态，牢固树立忧患意识、担当意识、进取意识，紧紧围绕发展目标，谋划好既利当前又利长远、既利经济增长又利环境保护、既利有效投入增加又利风险防范化解的思路举措，牢牢把握经济工作主动权。要保持经济运行稳定在合理区间，必须认真落实积极的财政政策和稳健的货币政策，着力强化消费的基础作用，实现社会消费品零售总额增长12%；继续发挥投资的关键作用，实现全社会固定资产投资增长13%、工业投资增长12%；大力发挥出口的支撑作用，实现进出口总额增长3%、出口增长5%；有效发挥金融的保障作用，保持信贷总量稳定增长，实现社会融资规模1600亿元，其中新增本外币贷款800亿元左右。

　　今年重点在以下五个方面狠下功夫。

　　（一）把“迈上新台阶、建设新无锡”鲜明地写在无锡发展的旗帜上

　　去年12月，习近平总书记视察江苏时发表了重要讲话，充分肯定了江苏经济社会发展和各项工作取得的成绩，明确了江苏为全国发展探路和“两个率先”的光荣使命，描绘了建设经济强、百姓富、环境美、社会文明程度高的新江苏美好蓝图，提出了“五个迈上新台阶”的工作要求，发出了“迈上新台阶、建设新江苏”的动员令，是指导江苏工作的总要求、总命题、总纲领。

　　做好今年政府工作，必须把贯彻落实好总书记视察江苏重要讲话精神作为最重要任务。要把“迈上新台阶、建设新无锡”鲜明地写在无锡发展的旗帜上，使之成为全市上下的共同思想基础和自觉追求，成为无锡发展的主旋律、最强音。全力推动经济发展迈上新台阶。关键举措是转方式调结构，坚持以提高经济发展的质量和效益为中心，大力实施创新驱动战略，积极发展先进制造业和现代服务业，切实加强生态环境保护。重要任务是有效防范和化解经济风险，强化“政银企”有效合作，着力化解中小企业融资担保链风险，努力实现不良贷款率继续下降；多措并举加速存量商品房消化，努力保持房地产市场健康发展；有效加强政府性债务管理，调整优化债务结构，严格控制新增债务，牢牢守住不发生区域性系统性风险的底线。全力推动现代农业建设迈上新台阶。虽然无锡农业占比较低，但基础性地位没有改变。必须严守耕地红线，确保基本农田面积不减少。着力解决“谁来种地”的问题，精心培育专业大户、家庭农场、专业合作社、农业龙头企业等新型农业经营主体和新型现代职业农民。深入推进无锡国家现代农业示范区建设，转变农业发展方式，提高农业基础设施和装备水平，积极发展精品农业、生物农业、生态农业、智慧农业、休闲观光农业等农业新兴产业，全市农业园区面积占耕地面积比重达45%以上。全力推动文化建设迈上新台阶。巩固全国文明城市创建成果，大力培育和践行社会主义核心价值观，继承发扬“四千四万”精神，学习身边好人，传承无锡优秀文化，增强文化凝聚力。积极创建国家公共文化服务体系示范区，实施文化惠民工程，发展先进文化，抵制有害文化，提高公共文化服务水平。扎实做好惠山祠堂群申遗工作。积极培育重点文化产业项目，大力推进国家数字电影产业园、无锡国家动画产业基地等产业园区建设，促进文化产业集聚发展、提质转型，实现文化产业增加值占地区生产总值比重达5%。全力推动民生建设迈上新台阶。按照守住底线、突出重点、完善制度、引导舆论的基本思路，着力抓好民生保障，扎实办好民生实事，努力让人民群众有更好的教育、更稳定的工作、更满意的收入、更可靠的社会保障、更高水平的医疗卫生服务、更舒适的居住条件、更优美的环境。全力推动全面从严治党迈上新台阶。把全面从严治党的要求落实到政府工作之中，以“要求严、措施严、对上严、对下严、对事严、对人严”的“六个严”标准，切实加强政府自身建设，坚决反对“为官不为”，使党的群众路线教育实践活动成果不断得以巩固和发展。

　　（二）把转方式调结构和生态文明建设放在更加重要位置

　　在当前经济下行压力较大的情况下，抓好经济发展是摆在无锡面前的一场硬仗。打好这场硬仗，关键是转方式调结构，同时把生态环境的倒逼压力转化为转型升级的自觉行动，努力走出一条经济发展和生态文明相辅相成的新路子。

　　大力实施创新驱动发展战略。深入开展苏南国家自主创新示范区建设。充分发挥自身优势，加强政策对接，学习借鉴中关村科技园政策经验，积极开展股权激励、税收优惠、科技金融等鼓励创新政策的先行先试，全面提高自主创新能力。突出企业创新主体地位，多渠道增加科技投入，全社会研发投入占地区生产总值比重达2.8%。加快推进国家传感网创新示范区建设。着力攻克一批物联网核心技术，培育壮大龙头骨干企业，累计建成省级以上物联网应用示范工程50项，物联网年营业收入超10亿元企业25家，打造无锡创新驱动的特色标志。加强智慧城市建设，实施一批重点应用工程，积极建设城市大数据中心。壮大战略性新兴产业规模。认真组织实施超级计算机、极大规模集成电路成套工艺等国家重大科研项目，加快推进云计算、微电子、节能环保、新能源及新能源汽车、新材料、生物医药、软件与服务外包等新兴产业发展，力争新兴产业产值增长15%，规模以上工业中战略性新兴产业占比达到30%左右。加大人才引进培养开发力度。营造开放包容的创新环境，进一步引进和用好高层次人才，深化与高等院校、科研院所合作，加强技能人才培养，鼓励本土人才脱颖而出。积极支持科技型中小企业做优做强，扩大规模产出效应。加强知识产权运用和保护，依法打击侵权行为，高标准建设国家知识产权示范城市。

　　大力发展先进制造业和现代服务业。立足百年工商城产业基础，弘扬“锡商精神”，着力打造面向全球的先进制造业基地和面向长三角乃至更广区域的现代服务业高地，增创产业发展新优势。扎实开展“项目建设突破年”活动。把重大项目作为带动转型升级的重要抓手，加快推进180个市级重大项目建设。围绕推动产业集聚，充分发挥现有500万平方米科研载体作用，着力推进重大项目招引，进一步加强与央企、沪企战略合作，实现产业链招商新突破，争取新增超10亿元或3000万美元产业项目70个。加快推进工业化和信息化深度融合。顺应新工业革命趋势，鼓励企业采用数字化、智能化制造模式，支持传统企业通过嫁接和运用信息技术实现提质增效，推动实施“机器换人”，进一步提高产业链整体效率，新增省级以上“两化融合”示范试点企业30家。下决心淘汰落后产能，做好产能过剩企业的关停并转工作。实施质量强市战略。推行卓越绩效管理，支持企业参与标准制订，加强质量攻关，争创“名品、名企、名牌”，进一步扩大宣传和影响，让“无锡品牌”“无锡质量”成为推动经济大市向经济强市迈进的有力支撑。着力打造新商贸业态中心。整合放大无锡区位、交通、信息技术和资源禀赋等优势，鼓励企业开展基于互联网的经营模式创新，提升各类电商服务平台功能，规范发展大宗商品电子交易，加快推进跨境电子商务发展。积极引进知名物流和仓储企业，推动电商、快递和智能仓储深度融合。加快建设华东航空快件集散中心，提升苏南快递产业园建设水平。培育发展现代都市服务业。在巩固和提升传统服务业的同时，加快发展信息服务、科技服务、现代金融和现代物流等生产性服务业，壮大教育培训、健康养老、人力资源服务、工业设计与文化创意、商务会展、体育产业等现代服务业规模，实现服务业增加值占地区生产总值比重提高1个百分点。提升拓展文化旅游产业。统筹锡澄宜文化旅游资源整合与联合开发，精耕细作传统旅游业，积极发展主题旅游、文化旅游、体验旅游、休闲旅游、乡村旅游等新兴旅游业，进一步提升游客满意度，实现游客人数和旅游总收入均增长10%。

　　大力促进开放型经济发展。主动对接国家重大开放战略。加强与上海自贸区的对接互动，鼓励企业到自贸区设立公司，促进投资、贸易便利化，加快形成一批可以全面推广的制度化成果。积极呼应“一带一路”和长江经济带建设，努力在互联互通、产业合作、商贸流通等方面取得实质性进展。一着不让抓好外资外贸工作。拓宽利用外资领域，加大高端制造、现代服务业、地区总部等引资力度，确保利用外资有所增长。奋力开拓国际市场，提高自主品牌和服务贸易出口比例，拓展外贸增长空间。加强口岸和功能载体建设。扩大口岸开放，新增一批指定口岸，积极推进设立无锡邮政口岸。完善综合保税区、经济开发区和特色园区功能，增强对高端产业综合承载力。深入实施“走出去”战略。鼓励企业到境外设立营销网络、研发机构，支持企业参与国际并购，推进境外经贸合作，提高全球范围资源配置和市场拓展能力。加大城市对外交流，提升无锡城市国际影响力。

　　大力推进生态文明建设。落实生态红线区域规划和主体功能区计划，实施生态补偿制度，切实加强环境治理和保护，集中力量解决影响群众生产生活的突出环境问题，全面完成“十二五”节能减排任务。加强大气污染防治。推广使用清洁能源，严格控制煤炭消费总量，提升燃煤锅炉高效清洁发展水平。推进工业废气治理，强化机动车尾气、建筑扬尘等污染防治，落实秸秆综合利用政策和禁烧措施，加强区域环境监测预警和应急处置，确保PM2.5平均浓度下降幅度达到国家考核要求。加强太湖水污染治理。加快国家水利现代化示范市和水生态文明示范市建设，实施太湖周边片区环境综合整治，推进新沟河拓浚、九里河及伯渎港整治等重点工程，强化“河长制”管理，加强饮用水源地保护和安全供水高速通道管理维护，确保饮用水安全、确保不发生大面积湖泛。加强生活环境整治。继续推进生活垃圾分类收集、分类处置试点，完善收运体系建设，增加终端处置能力，有效化解垃圾增长与处理能力的突出矛盾。提高固体废弃物处置能力，开展重金属污染物等专项整治和土壤修复试点。深入推进绿色无锡建设，新增造林绿化面积1.1万亩，林木覆盖率26.7%以上。加强环保执法监管。严格执行新《环境保护法》，坚持铁腕治污、依法治污，强化行政执法与司法联动机制，大力开展环保执法专项行动，推进环境保护从污染治理向全过程监管转变。

　　（三）把新型城镇化和民生保障的事情办好办实

　　突出以人为本理念，顺应群众对美好生活的新期盼，找准民生改善与经济发展的结合点、发力点，促进经济建设与新型城镇化、民生改善良性互动，切实办好12件80项民生实事项目，让人民群众在改革发展中普遍得益受惠。

　　扎实推进新型城镇化。强化“一体两翼”互联互通。统筹规划建设锡澄、锡宜重大交通基础设施，加快建设宜兴通用机场，推动沿江城际铁路开工建设，做好泰锡宜铁路及过江通道、新长铁路扩能改造、苏锡常南部高速无锡段等前期工作，加快新锡澄路等地方干线公路建设。开工建设锡澄运河航道整治工程市区段，启动锡溧漕河二期整治工程。推进无锡（江阴）港功能完善和服务能力提升。着力提高中心城区能级。理顺体制机制，整合资源配置，优化商贸商务服务，增添都市产业功能，巩固提升商业中心地位。加快老城区改造步伐，加强背街小巷整治和保洁，抓好重点片区、重要路段的市容环境美化，塑造具有城市个性的建筑文化和空间特色。加快“一城一岛一带”等重点区域开发。完善太湖新城产业布局和配套功能设施，加快马山岛休闲旅游项目建设，加强古运河及其周边片区开发中的历史文化保护，提升锡东新城、惠山新城、江阴临港开发区、宜兴东氿新城集聚产业和人口能力。增强城乡基础设施功能。加快建设民航无锡空中交通管理站，基本建成沪宁城际铁路惠山客运站，做好锡太高速前期工作。实施钱胡路等城市道路改造，改善重点片区道路通行条件。有序推进地铁3号线一期、1号线南延工程建设，提高地面公共交通与地铁接驳换乘能力，新辟、优化公交线路15条。推进农村公路维护和提档升级。新建500千伏宜兴东、陆桥等输变电项目，完成西区燃气热电项目主体工程建设。改造老旧自来水管网60千米，新增天然气用户6万户，增设“苏邮惠民”便民服务站300家，免费无线接入点达4万个以上。统筹城乡一体化发展。推进城市总体规划修编，优化镇村布局规划，加强园区布局与城镇建设有机衔接。构建节约集约用地战略布局，科学调整科研及产业用地性质，创新推进城镇低效用地再开发。积极推动公共服务向农村延伸，加快城乡社会保障一体化。推进城乡一体化先导示范镇建设，打造一批重点镇和特色镇。加强历史文化名村的修缮保护，建设一批具有江南水乡特色的美丽乡村、休闲旅游示范村。

　　务实推进民生改善。提高居民收入和就业保障水平。努力拓宽增收渠道，重视增加城乡居民财产性收入。全面实施更加积极的就业创业政策，大力支持城乡居民自主创业，新增城镇就业10万人以上，扶持自主创业1万人以上。实施全民参保登记计划，全市养老、医疗保险分别扩面6万人，失业、工伤和生育保险分别扩面4万人，继续合理调整城乡居民基础养老金标准。城乡社区居家养老服务实现全覆盖，为2.5万户困难家庭老人提供居家养老援助服务。统筹各类社会救助资源，完善临时救助制度，推进红丝带关爱中心建设。改善群众居住条件。用好国家棚户区改造政策，实施20万平方米危旧房改造，完成60个城中村、191万平方米旧住宅区综合整治。在做好村庄规划基础上，规范农村住房建设管理。加强安置房、经适房、老小区等社区物业管理，继续推进老小区电梯和技防改造。加强市场建设和流通领域监管。强化食品药品监管，食品、药品安全检测合格率分别达到97.5%和99%。抓好市场保供稳价，建立价格惠民常态体系，健全市区142家平价商店监管服务网络平台。市区新建、改造农贸市场12家。建立完善城市公共配送体系，提高商品流通效率。

　　加快推进社会事业发展。提高教育现代化建设水平。优化发展基础教育，加快培育建设新优质学校，支持农村学校改善办学条件，深入实施素质教育，全面提高义务教育学校现代化办学水平。加快市少年宫和一批新办学校建设，新建、改扩建幼儿园30所，不断完善社会化早教体系。积极引进优质本科教育资源，加快建设江南大学无锡医学院，推动省市共同支持无锡太湖学院建设。加快发展现代职业教育，支持各类职业院校提高办学质量，深化与产业转型融合发展。提高医疗卫生服务水平。切实改善医疗服务质量，着力解决群众看病就医难题。加强基层公共卫生机构建设和运行管理，壮大全科医生队伍。强化医疗人才培养和学科、专科建设，新增一批国家级、省级重点专科。完善急救站点城乡一体化建设，开展健康促进和慢性病干预。提高体育事业发展水平。贯彻全民健身国家战略，争创全国公共体育服务体系示范区。引进更多国际精品赛事，办好环太湖国际马拉松赛、公路自行车赛等一批重大体育赛事。积极发展青少年校园足球。

　　进一步推进社会治理创新。深化社区扁平化管理。巩固社区减负成果，规范社区事务准入。创新基层管理服务，加强社会工作人才队伍建设，积极培育社会组织，充分发挥各类基层组织、社会团体和志愿者作用，推进政社互动和社区多元共治，建设和谐文明城乡社区。预防化解社会矛盾。推进落实重大决策社会稳定风险评估，畅通群众诉求表达渠道。全面排查各类矛盾纠纷，妥善处理好信访热点和遗留问题，把涉法涉诉信访纳入法治轨道解决。依法做好征收拆迁工作，着力破解征收拆迁难题。加强公共法律服务体系建设，完善司法救助制度。努力打造诚信无锡。健全社会信用体系，强化守信激励和失信惩戒机制，加大打击逃废债力度，让“诚信无锡”和“法治无锡”相得益彰。加强平安无锡建设。深化完善立体化、现代化社会治安防控体系，严厉打击各类违法犯罪行为。强化网络信息安全管理。加强安全生产工作，进一步夯实基层基础，严格责任落实，强化隐患治理和监管执法，确保不发生重特大安全事故。健全应急管理机制，提高公共安全和防灾减灾能力。认真做好台港澳、侨务、民族宗教等工作。深入开展国防教育，加强国防动员和双拥工作，提高军地融合发展水平。

　　（四）把全面深化改革和全面推进法治建设贯穿于政府工作全过程

　　坚持深化改革和法治建设“两个轮子”一起转，一方面扎实开展“改革创新突破年”活动，加大重要改革推进力度，推动各项改革举措尽快落地见效；另一方面，切实发挥法治的引领和规范作用，自觉运用法治思维和法治方式来深化改革开放、规范发展行为、促进矛盾化解、维护社会稳定。

　　继续推进全面深化改革。深化行政体制改革。积极稳妥推进政府机构改革，强化机构和职责整合，规范机构设置，完善体制机制。进一步规范行政审批行为。健全“一办三中心”政务服务平台功能，加快网上政务服务系统建设。实施工商营业执照、组织机构代码证和税务登记证“三证合一”登记制度。全面推行权力清单和责任清单管理，加强事中事后监管。深化财税体制改革。推行全口径预算管理，严格财政支出，依法清理规范土地、税收等优惠政策。适度调整市对区财政体制，完善镇、街道财力保障机制。探索实行政府和社会资本合作（PPP）模式。深入推进 “营改增”试点，认真落实国家扶持企业发展的结构性减税政策。深化国有企业改革。鼓励发展混合所有制经济，支持各类资本参与国企改制重组，加快国有资本证券化步伐。完善现代企业制度，加强内控机制建设，提高重大决策、资本运作规范化水平。试行职业经理人制度，完善薪酬管理和激励约束机制。深化金融领域改革。大力发展产业金融，积极推进多层次金融市场建设。加快发展地方金融机构，完善地方金融控股集团机制，支持民间资本发起设立民营银行，争取设立消费金融公司、金融租赁公司，促进互联网金融、融资性担保公司加快发展。鼓励企业上市和发行债券，推动上市企业兼并重组。扩大金融对外开放，拓展外汇管理便利化改革试点，推进无锡海峡两岸金融与科技合作试验区建设。深化农村领域改革。启动农村土地承包经营权抵质押融资试点，抓好农村土地承包经营权、农民宅基地及住房、农村集体资产确权登记颁证工作，稳妥推进农村土地征收、集体经营性建设用地入市、宅基地和集体产权制度改革试点。深化社会事业改革。调整完善“管办分离”体制。完善教育评价制度，健全教师激励机制，探索建立中小学校长职级制，按照国家和省统一部署实施考试招生制度改革。推进城市公立医院改革，加快区域医疗联合体建设，引导优质医疗资源下沉社区，健全重特大疾病医疗保险和救助体系。启动实施机关事业单位养老保险制度改革。推进国家养老服务业综合改革试点，实施公办养老机构改革，促进医养融合发展。鼓励更多社会力量参与教育、卫生、养老和社区服务。继续深入开展全国流动人口社会融合试点工作，完善居住证管理制度。深化水电气等资源环境价格改革。

　　全面推进依法行政。健全依法决策机制。完善重大事项决策的立项调研、公众参与、专家论证、风险评估、合法性审查、集体讨论决定等程序，加强政府法律顾问制度建设，建立重大行政决策责任追究制度。提高依法行政能力。严格依法办事，规范行政权力运行，做到法定授权必须为、法无授权不可为。落实行政执法责任制，按照职权法定、权责一致的原则，规范行政执法主体，明晰职责权限，切实解决执法不严、违法不究等问题。强化行政权力制约监督。自觉接受人大及其常委会的法律监督、工作监督，自觉接受政协民主监督，规范重大决策出台前向人大常委会报告、向政协通报制度。适应行政诉讼制度新变化，自觉接受司法监督。重视公众监督、舆论监督，依法保障群众和新闻媒体直接监督政府的权利。强化审计监督，对公共资金、国有资产、国有资源和领导干部经济责任履行情况，实现有重点、有深度、有步骤、有成效的审计全覆盖。营造全社会法治氛围。引导广大市民自觉守法、遇事找法、解决问题靠法，完善调解、仲裁、行政裁决、行政复议等多元化纠纷解决机制。加强社会普法宣传，推动普法宣传进机关、进乡村、进社区、进学校、进企业、进单位。积极推动公正司法建设，努力让群众在每一个司法案件中感受到公平正义。

　　（五）切实加强服务型政府和廉洁政府建设

　　打铁还需自身硬。我们将切实增强责任感和使命感，深入开展“作风建设突破年”活动，进一步增强服务理念，加强廉洁自律，努力建设人民满意的服务型政府和廉洁政府。

　　着力加强服务型政府建设。严格执行中央八项规定和省、市委十项规定，进一步改进文风会风，严格控制节庆、论坛、展会及评比表彰等活动，严格压缩“三公”经费支出，完成机关公务用车改革。提高行政服务效能，强化政府绩效管理、效能监察和行政问责，大力整治行政不作为、慢作为、乱作为现象，打通服务群众“最后一公里”，提高政府执行力和公信力。深入开展领导干部下基层“三解三促”活动，广泛开展机关干部进社区活动，尽力帮助基层和群众排忧解难。进一步完善“稳心留根”机制，开展“暖企行动”，使广大企业切身感受到政府优质服务的温暖。深入推进政务公开，加强公共资源配置、重大建设项目、社会公益事业、审计结果等重点领域信息公开，做好政府组成部门预决算和“三公”经费预决算公开工作。建立重要政府信息定期发布机制，完善新闻发言人制度，充分发挥政府网站主渠道作用，及时回应社会关切。

　　着力加强廉洁政府建设。严格落实党风廉政建设责任制，着力构建政府系统惩防体系，深入开展警示教育和廉政文化建设，加强对政府内部权力制约，落实好“一岗双责”制度，从源头上预防和治理腐败。坚持以零容忍态度惩治腐败，严肃查处发生在重点领域、关键环节和群众身边的腐败案件，坚决纠正征收拆迁、环境保护、工程建设、教育卫生、行政执法等领域损害群众利益的不正之风，努力以实实在在的成效取信于民，树立为民务实清廉的政府形象。

　　今年还有一项重要工作，就是编制“十三五”发展规划。我们将充分发扬民主，凝聚各方智慧，深入调查研究，以改革创新的精神，努力编制好一个适应时代要求、体现中央精神、符合无锡实际、反映群众意愿的“十三五”规划。

　　各位代表，展望新的一年，我们面临的任务艰巨而繁重，肩负的责任重大而光荣。让我们紧密团结在以习近平同志为核心的党中央周围，在省委、省政府和市委的正确领导下，开拓创新，务实奋进，全面完成今年经济社会发展目标任务，为建设经济强、百姓富、环境美、社会文明程度高的新无锡而努力奋斗！
